# Low PSI content broadens the optimal light spectrum for a phycoerythrin-dominated cyanobacterium towards near far-red

**DOI:** 10.1038/s41598-026-50772-z

**Published:** 2026-05-07

**Authors:** Mariann Kis, Tomáš Zavřel, István Fodor, Anna Segečová, Péter Urbán, Bence Gálik, Róbert Herczeg, Attila W. Kovács, László Kovács, Martin Lukeš, Gábor Bernát

**Affiliations:** 1 HUN-REN Balaton Limnological Research Institute, Tihany, 8237 Hungary; 2 Global Change Research Institute, CAS, 603 00 Brno, Czech Republic; 3 HUN-REN Biological Research Centre, Szeged, 6726 Hungary; 4 Genomics and Bioinformatics Core Facilities, Szentágothai Research Centre, University of Pécs, Pécs, 7624 Hungary; 5 Centre Algatech, Institute of Microbiology, 379 01 Třeboň, Czech Republic

**Keywords:** Cyanobacterial photosynthesis, Chromatic acclimation, Fluorescence, Pigment composition, Ecology, Ecology, Plant sciences

## Abstract

Light quality is an important yet often overlooked driver of cyanobacterial physiology, ecology, and productivity. Here, we characterize the spectral acclimation strategies of cyanobacterium *Cyanobium* sp. NIVA-CYA 375 across the full photosynthetically active radiation range, using narrow-band LEDs with distinct emission profiles. Genomic analysis confirmed that, despite the presence of *cpeA* and *cpcL* genes, *Cyanobium* lacks chromatic acclimation capacity and functions as a spectral generalist. Biophysical and biochemical measurements revealed that this strain possesses high phycoerythrin content in its phycobilisomes (PBS), and inherently low photosystem I (PSI) content relative to photosystem II (PSII). Although phycoerythrin absorbs green light efficiently, *Cyanobium* exhibited chronic acceptor-side limitation of PSII, constraining its electron transport capacity under most wavelengths including green. Notably, the highest growth rates occurred under near far-red light, due to simultaneous excitation of PSI via chlorophyll *a* and PSII via phycobilisomes. Despite attempts to balance electron transport rates by increasing absolute PBS abundance and/or PBS attachment to PSII or PSI, other cultivation wavelengths resulted in insufficient excitation of either PSII or PSI, presumably imbalancing NADPH and ATP formation. Our results suggest that low PSI content is an adaptive trait allowing *Cyanobium* to thrive in the spectrally variable, low-light conditions of turbid freshwater habitats. These findings reveal how low PSI content can drive spectral niche specialization and shape the wavelength-dependency of growth in cyanobacteria.

**Supplementary Information:**

The online version contains supplementary material available at 10.1038/s41598-026-50772-z.

## Introduction

Cyanobacteria are a large and diverse group of primary producers that inhabit marine, freshwater, and terrestrial environments. Due to their global distribution and abundance, they represent one of the most ecologically significant groups of primary producers on Earth. Their success in colonizing nearly all environments is attributed to their minimal nutritional requirements and their ability to flexibly adjust cellular metabolism to diverse ecological niches and fluctuating conditions^1^. Cyanobacteria perform oxygenic photosynthesis, a light-driven process in which water serves as the primary electron donor when oxidized to molecular oxygen. Electrons are transferred through the photosynthetic electron transport chain (PSET) and ultimately reduce NADP^+^ to NADPH. The energy conserved as ATP and the reducing power stored in NADPH are subsequently used to assimilate atmospheric CO_2_ into energy-rich carbohydrates.

As obligate phototrophs, cyanobacteria must sense and precisely respond to many environmental factors to optimize photosynthetic efficiency. The key components of the cyanobacterial photosynthetic machinery are the phycobilisomes (PBS) and the two chlorophyll-associated reaction center complexes, photosystem I (PSI) and photosystem II (PSII), which together capture and convert light energy into chemical energy^2^. More than 100 chlorophyll (Chl) variants have been identified to date, with Chl *a* being the predominant form in cyanobacteria^3,4^. Despite their diversity, all chlorophylls share a similar core structure and display strong absorption in the blue and red regions of the visible spectrum. Carotenoids act as accessory light-harvesting pigments and also play key photoprotective roles by dissipating excess excitation energy as heat and scavenging reactive oxygen species^5,6^.

Chlorophylls and carotenoids absorb only weakly in the 500–650 nm region of the visible spectrum. PBS, large peripheral antenna complexes with characteristic spectral properties, partially compensate for this absorption gap^7^. PBSs consist of two main substructures—the core and the rods—and are built from phycobiliproteins (PBPs) that bind bilin chromophores to absorb light, and linker proteins that organize the subunits and anchor the complex to PSII or PSI^8–10^. PBPs are classified into four major classes: allophycocyanin (AP; λ_max_ = 650–665 nm), phycocyanin (PC; λ_max_ = 615–640 nm), phycoerythrocyanin (PEC; λ_max_ = 575 nm), and phycoerythrin (PE; λ_max_ = 565–575 nm)^11^.

Light quality has been recognized as one of the key factors driving the global distribution of phytoplankton^12^. To optimize light capture across diverse underwater spectral niches^13^, cyanobacteria have evolved mechanisms to adjust the composition of their PBS through a process known as chromatic acclimation (CA). To date, seven distinct CA types have been described, designated CA1–CA7^14,15^. CA1 features a green light-dependent association of CpcL-containing PBS with PSI and the formation of canonical PBS under red light^16^. During CA2, PE synthesis is upregulated under green light. CA3 involves complementary pigment adjustments, with PE upregulated under green light and PC upregulated under red light. During CA4, PBS chromophore composition is modified, shifting phycourobilin and phycoerythrobilin levels in response to blue and green light without altering PBS protein structure. CA5 and CA6 are associated with acclimation to red and far-red light. In CA5, light harvesting is mediated by Chl *d* in the thylakoid membrane and PBS, the presence of which may be strain-dependent^17^. CA6 triggers PBS remodeling induced under far-red light, which includes the production of far-red shifted allophycocyanin and alternative photosystem components, along with a shift from Chl *a* to Chl *d*/*f*^15^. CA7 enhances the absorption of yellow-green wavelengths by modulating phycoerythrocyanin levels^14^. Notably, many cyanobacteria can perform hybrid CA types, such as CA1/7, which regulates rod-shaped phycobilisomes and phycoerythrocyanin levels, and CA1/3, which regulates rod-shaped phycobilisomes and PE/PC ratios^14,18^.

In addition to CA-capable strains, many cyanobacteria are referred to as green- or blue-light specialists. These strains maintain a fixed pigment composition, which provides a competitive advantage in environments dominated by green or blue wavelengths^19^. In contrast, spectral generalists are characterized by their ability to utilize a broad range of light to drive photosynthesis—not through inducible CA, but via a constitutively broad pigment repertoire and flexible regulation of photosystem stoichiometry. More generally, cyanobacterial light acclimation operates on multiple levels and timescales to fine-tune linear, cyclic and respiratory electron flows. Rapid, reversible responses such as state transitions and non-photochemical quenching (NPQ) protect the photosystems^20,21^, while slower regulatory layers, including, activation of futile cycles, reactive-oxygen-species scavenging and global transcriptomic or proteomic remodeling secure acclimation to persistent changes in light quality or intensity^22–26^. Additionally, the PSII-to-PSI ratio or the functional attachment of PBS to PSII or PSI can be adjusted, leading to shifts in photosynthetic, cyclic and respiratory electron transport rates, which ultimately lead to alterations in cellular composition^27–29^.

In this work, we study the light-color acclimation mechanisms in the unicellular picocyanobacterium *Cyanobium* *sp.* NIVA-CYA 375 over the entire photosynthetically active radiation (PAR) spectrum, ranging from 435 to 687 nm. We systematically analyzed the long-term acclimation strategies of this microorganism, focusing on changes in pigment composition, light-harvesting properties, and excitation-energy distribution. This strain has PE-rich PBS, gaining a fitness advantage under the green part of the PAR spectrum. However, we found that it grows equally well under red light, with the optimal growth occurring in the near far-red region. We show that *Cyanobium* *sp.* NIVA-CYA 375 has low PSI content, which limits its growth under most cultivation wavelengths tested. Similar to other cyanobacteria, growth was the most limited under blue light, due to the low probability of light capturing and using the absorbed photons for photochemistry. On the other hand, cultivation under near far-red (687 nm) light allowed for the fastest growth rate within the PAR spectrum. We show that this was related to the simultaneous excitation of both PSII via PBS and PSI via Chl *a*. Our results with *Cyanobium* sp. NIVA-CYA 375 thus reflect the specific light requirements of cyanobacteria with low PSI content. Growth under 687 nm light was higher than under green light, where PE absorbs the most. This indicates that low PSI content can shift the optimal growth wavelength beyond the spectral region predicted by the dominant PBS pigments alone, extending efficient photosynthesis into the near far-red range. For cyanobacteria living in turbid shallow lakes—where red and near far-red photons are relatively enriched—this shift may represent a significant ecological advantage.

## Materials and methods

### Strain and cultivation conditions

The strain *Cyanobium* sp. NIVA-CYA 375 (denoted as *Cyanobium* hereafter) was isolated from Lake Balaton, Hungary, and has been deposited as strain ACT 9807 at the Algal Collection Tihany, Hungary. Its 16S rRNA nucleotide sequence shows similarity to Subalpine I group strains^30^. *Cyanobium* cultures were grown in Erlenmeyer flasks at 24 °C in liquid BG-11 medium^31^ under ambient air in a batch regime, with periodic culture dilution preventing stationary phase. The stock cultures were cultivated under warm-white fluorescent lamps at a photon flux density (PFD) 25 μmol photons m^−2^ s^−1^. During the light quality acclimation experiments, the cultures were illuminated by monochromatic light emitting diodes (LEDs) with peak wavelengths ranging from 405 to 687 nm. Illumination was provided as diffuse light from below the Erlenmeyer flasks on a home-built cultivation apparatus; the LED specifics have been described previously^27,28^. The light regime was set to 14:10 (hours of light:hours in dark) and mixing was provided on a daily basis to prevent cell sedimentation.

### Fluorescence kinetic measurements

Activity of PSII in *Cyanobium* was probed by a Multi-Color PAM (Walz, Germany) and AquaPen (Photon Systems Instruments, Czechia). The Multi-Color PAM was used to record the functional absorption cross-section of PSII (σ_II_) and slow Chl *a* fluorescence kinetics (SP-Analysis mode). σ_II_ was measured with the default Multi-Color PAM script Sigma1000cyano, default trigger file Sigma1000 and a default fitting protocol described in detail previously^32^. The σ_II_ values were used for the calculation of electron transport rate (ETR(II)) based on the quantum absorption and yield of PSII:

1 $$ETR(II) =PAR(II) \times \frac{Y(II)}{{Y(II)}_{max}}$$

2 $$PAR(II) ={\sigma}_{II} \times L \times PAR$$

where PAR is PAR intensity , PAR(II) is the rate of PAR absorption by PSII, L is Avogadro’s constant, ETR(II) is the rate of electron transport at PSII, Y(II) is the effective PSII quantum yield under actinic light (AL) and Y(II)_max_ is the PSII quantum yield in the dark-acclimated state under which σ_II_ was determined^32^. Y(II), used for ETR(II) calculation, was recorded during the slow Chl *a* fluorescence kinetic measurements, as:

3 $$Y(II) = \frac{{F}_{m}^{\prime} - {F}_{t}}{{F}_{m}^{\prime}}$$

where F′_m_ and F_t_ are maximal and steady-state fluorescence intensities under actinic light (AL), respectively. Y(II) and ETR(II) were determined under 440 nm and 625 nm AL, both with PFD 100 μmol photons m^−2^ s^−1^, inducing *State 1* and *State 2*, respectively^20^. This also allowed us to monitor state transitions, as a difference in F′_m_ values after switching the AL source.

To induce NPQ, AL of 480 nm with PFD 1 500 μmol photons m^−2^ s^−1^ was additionally applied for 2.5 min. NPQ was calculated from maximal fluorescence value in steady-state under high AL (F′′_m_) and during the entire course of each measurement (F′_m__(max)_)^33^:

4 $$NPQ = \frac{{\left( {F^{'} _{{m\left( {max} \right)}} - F^{{''}} _{m} } \right)}}{{F^{{''}} _{m} }}$$

OJIP curves were recorded in light-acclimated *Cyanobium* cultures, using AquaPen (Photon Systems Instruments, Czechia) and a 620 nm saturation pulse of PFD 3 000 μmol photons m^−2^ s^−1^. From the OJIP curves, the following parameters were derived:

5 $${\varphi {P}_{0}}= \frac{ {F}_{M}- {F}_{O}}{{F}_{M}}$$

6 $${V}_{J} = \frac{{F}_{J} - {F}_{O}}{{F}_{M} - {F}_{O}}$$

7 $${V}_{I} = \frac{{F}_{I} - {F}_{O}}{{F}_{M} - {F}_{O}}$$

8 $${\psi E}_{0} = 1-{V}_{J}$$

9 $${\psi R}_{0} = 1-{V}_{I}$$

10 $${\varphi R}_{0} = \varphi {P}_{0}*{\psi R}_{0}$$

where F_O_, F_J_, F_I_ and F_M_ refer to the fluorescence yields at the O, J and I points of the OJIP curve, and the maximum fluorescence yield, respectively. The parameter φP_0_ represents the maximum quantum yield of PSII, V_J_ represents a proxy of the redox state of the plastoquinone pool^34,35^, ψE_0_ and ψR_0_ represent the efficiency with which a PSII-trapped electron is transferred to PQ or PSI, respectively, and φR_0_ represents overall quantum yield of electron transport from PSII to PSI^36,37^. Fluorescence data were pre-processed by an in-house-developed data processing tool available at [https://tools-py.e-cyanobacterium.org/].

### PSI activity measurements

P_700/_P_700_^+^ oxidation/re-reduction kinetics were recorded after 10 min of dark acclimation using a Dual-PAM-100 measuring system (Walz, Effeltrich, Germany) set to “FastAcquisition” mode. Culture aliquots were filtered through glass fiber filters (GF/B, Whatman). Wet filters were placed between two microscope glass slides embedded in a DUAL-B leaf holder^28^. The P_700_^+^ re-reduction rates (*k*; units s^-1^) were determined by fitting the 830 nm absorption signal decay kinetics in dark, following PSI oxidation by SP (635 nm, 100 ms), with a single-parameter exponential function^29^.

### UV–Vis absorption spectroscopy

The steady-state absorption spectra of the cultures were recorded at room temperature using a double beam spectrophotometer (Specord 210 Plus, Analytik Jena, Germany). The baselines were corrected for light scattering by placing four slices of tracing paper in front of both sample and reference cuvettes. The recorded baseline-corrected whole-cell spectra were used for calculation of photosynthetically usable radiation (PUR) for each narrow-band LED^28^:

11 $$PUR{}_{400}^{750} = {\int}_{400}^{750}(PAR\times abs)$$

where PAR represents photosynthetically active radiation spectra of the cultivation LEDs, and *abs* represents absorbance spectra of the corresponding *Cyanobium* cultures, respectively, in the wavelength range 400–750 nm. To determine growth rates, OD_750_ values were fitted by a modified Gompertz equation^38,39^**.**

### Steady-state low temperature (77 K) fluorescence spectroscopy

5 mL culture aliquots were filtered through a 25 mm GF/B glass microfiber filter (Whatman, UK), flash-frozen in liquid nitrogen and stored at -80 °C. For measurement, a slice from each filter was cut to fit the PMU-130 sample holder (Jasco, Japan) and immersed into a Dewar flask with a bottom transparent finger filled with liquid nitrogen. A series of 77 K fluorescence emission spectra, creating a 3D excitation-emission map, were recorded using a Jasco FP8550 spectrofluorometer with excitation and emission ranges 350–650 nm (step: 5 nm) and 500–800 nm (step: 0.5 nm), respectively. Bandwidth was set to 5 nm, scan speed to 1 000 nm min^−1^, and sensitivity to low. To distinguish Chl *a* fluorescence originating in PSII (Chl-PSII) or PSI (Chl-PSI) as well as phycobilisome functionally attached to PSII (PBS-PSII), to PSI (PBS-PSI) and PBS functionally uncoupled from both photosystems (PBS-free), equations summarized in a previous work were used^28^:

12 $$Chl-PSII = \frac{Ex{}_{440} Em{}_{689}}{Chl-total}$$

13 $$Chl-PSI = \frac{Ex{}_{440} Em{}_{724}}{Chl-total}$$

14 $$PBS-PSII = \frac{Ex{}_{560} Em{}_{689} + Ex{}_{620} Em{}_{689}}{PBS-total}$$

15 $$PBS-PSI = \frac{Ex{}_{560} Em{}_{724} + Ex{}_{620} Em{}_{724}}{PBS-total}$$

16 $$PBS-free = \frac{Ex{}_{560} Em{}_{580} + Ex{}_{560} Em{}_{662}+ Ex{}_{620} Em{}_{662}}{PBS-total}$$

17 $$Chl-total = Ex{}_{440} Em{}_{689} + Ex{}_{440} Em{}_{724}$$

18 $$\begin{aligned} PBS - total & = Ex_{{560}} Em_{{580}} + Ex_{{560}} Em_{{662}} + Ex_{{620}} Em_{{662}} + Ex_{{560}} Em_{{689}} \\ & \quad + Ex_{{620}} Em_{{689}} + Ex_{{560}} Em_{{724}} + Ex_{{620}} Em_{{724}} \\ \end{aligned}$$

where Ex and Em represent pigment fluorescence excitation and emission, respectively, and the subscript indexes represent corresponding wavelengths. The fluorescence data were pre-processed by an in-house-developed data processing tool available at [https://tools-py.e-cyanobacterium.org/ex_em_spectra_analysis].

### Cell composition and size

Relative cellular lipid, carbohydrate and protein content was determined by Fourier-transformed infrared spectroscopy (FTIR). 1 mL of the cell suspension was centrifuged (4000 × *g*, 5 min, 4 °C), the pellet was freeze-dried and analysed by a Nicolet IS10 FTIR spectrometer (Thermo Fisher Scientific, Waltham, MA, USA), following a previously described protocol^40^. The cellular content of glycogen, Chl *a* and total carotenoids as well as PBS was determined using previously described protocols^41–43^. To quantify individual carotenoids, additional analysis was performed by high-performance liquid chromatography (HPLC) following the method described in detail previously^27^. Cell size was determined by an ImageStream MkII imaging flow cytometer (Amnis Corp., USA) using a previously described method^26^.

### Genome sequencing and assembly

DNA was isolated using the Quick-DNA™ Miniprep Kit (#D3025, Zymo Research) following the manufacturer’s instructions and quantified using a Qubit 3.0 fluorometer (ThermoFisher). Library preparation was carried out using the xGen DNA EZ Library Prep Kit (#10009821, IDT). Briefly, 400 ng of genomic DNA was fragmented, end-repaired, adapter-ligated, and size-selected with magnetic beads to obtain 250–300 bp inserts. Finally, the library was amplified according to the manufacturer’s instructions. Library quality was assessed on a 4200 TapeStation System using D1000 ScreenTape (#5067–5582, Agilent), and concentrations were measured with Qubit 3.0. Illumina sequencing was performed on a NovaSeq X Plus platform using a 2 × 151 bp run configuration.

Raw sequencing reads were quality-filtered using FastQC and fastp with default settings, removing low-quality bases and adapter sequences to ensure high-quality input for downstream analyses. De novo assembly was performed with SPAdes (v3.15.0) using default parameters, including k-mer optimization, error correction, and iterative contig construction. Assembly completeness and accuracy were assessed with BUSCO (v5.7.1). The generated genomic data have been deposited in the NCBI BioProject database under the accession numbers PRJNA1377438 and SAMN53770833. Basic metrics are provided in Supplementary Table S1. Detailed CA type search was carried out using the workflow presented below.

## Reference genome selection and database construction

To establish a comprehensive reference dataset for CA-related genes in cyanobacteria, a set of well-annotated RefSeq genomes assembled at chromosome level was selected (406 genomes altogether). Only high-quality assemblies with curated annotations were considered in order to reduce potential artifacts arising from incomplete assemblies or fragmented gene models. The selected genomes were downloaded from the NCBI RefSeq database using the NCBI *datasets* command-line interface. For each genome, the protein sequences (proteome) and associated annotation files were retrieved. The individual proteomes were subsequently merged into a single FASTA dataset representing the reference cyanobacterial protein database used for downstream analyses. This combined dataset served as the primary sequence database for identifying homologous CA genes.

### Retrieval of CA genes

A set of genes involved in cyanobacterial CA systems was targeted based on the reference gene sets described previously^14,15,44,45^. The complete list of analyzed genes and their associated CA categories is provided in Supplementary Table S2. Because several CA genes are inconsistently annotated in public databases, a two-step strategy was applied consisting of 1) reference-guided homology searches, and 2) annotation-based sequence retrieval. This approach allowed reliable detection of CA-associated genes despite heterogeneous annotation practices across cyanobacterial genomes.

#### Manual reference sequence selection

For genes with inconsistent or unreliable annotation across public databases, representative reference protein sequences were manually selected from curated protein repositories (UniProt or NCBI Protein). For each gene, one to three high-confidence reference sequences were chosen and used as query sequences for homology searches.These reference sequences served as seed queries for BLAST-based similarity searches against the combined cyanobacterial proteome database described above. The reference-guided searches targeted genes including regulatory components (*ccaR, rcaC, rcaF, rfpA, rfpB, rfpC*), CA island genes (*fciA, fciB, fciC, mpeW, mpeZ*), selected phycobilisome linker proteins (*cpcA2, cpcB2, cpcG1, cpcG2*) and phycoerythrin rod linker proteins (*cpeC, cpeD, cpeE*). Homologous proteins were identified using BLAST-based similarity searches against the cyanobacterial reference proteome dataset. Searches were performed using standard BLAST parameters, including the BLOSUM62 substitution matrix, default low-complexity filtering, and an E-value threshold of 10. Candidate homologs were extracted from BLAST output tables based on their sequence identifiers and compiled for downstream analysis.

#### Annotation-based sequence extraction

For genes with consistent annotation across reference genomes, sequences were retrieved directly from the downloaded proteomes using gene identifiers present in the genome annotation files. This approach enabled straightforward extraction of protein sequences corresponding to specific gene names without the need for additional similarity searches. Genes primarily retrieved through annotation-based extraction included *pecC* and related phycobilisome linker components as well as additional structural phycobiliprotein-associated genes present in the selected reference genomes.

#### Construction of gene-specific sequence datasets

For each target gene, the identified homologous sequences were collected into gene-specific FASTA datasets. Duplicate or identical sequences were removed where necessary to generate representative sequence collections for each gene family. These curated datasets constituted a reference database of cyanobacterial CA-related genes suitable for comparative genomic analyses. The assembled genome of *Cyanobium* sp. NIVA-CYA 375 was subsequently screened for the presence of these CA-associated genes. BLAST results were filtered using the following thresholds: percentage identity: pident > 80 and alignment length: length > subject sequence length * 0.50. Genes were classified as present or absent (Y/N) based on these criteria (Supplementary Table S2).

#### Statistical analysis

Statistical analyses were conducted according to the workflow described previously^14,28^. All tests were performed using R Statistical Software^46^. When both the assumptions of normality and homogeneity of variances were satisfied^47^, ANOVA followed by Tukey’s HSD post hoc test was used. When only data normality was satisfied, Welch’s one-way ANOVA was used, followed by pairwise t-tests with Benjamini–Hochberg (BH) correction. The number of replicates was 3–4 for all growth lights throughout all experiments except for the cell composition parameters (n = 11–12). The *p* value threshold was set to 0.05.

## Results

### *Cyanobium* sp. NIVA-CYA 375 is not a CA-capable cyanobacterium

Homology searches using known genetic markers of chromatic type 1–7 genes revealed the presence of *cpcL* and *cpeA* genes in the genome of *Cyanobium* sp. NIVA-CYA 375 (Supplementary Tables S1, S2; Supplementary Figs. S1, S2). These genes encode the CpcL linker protein and the PE α-subunit, respectively. However, the absence of the corresponding photosensory systems (e.g., *ccaS/ccaR, rcaE, rfpA*; Supplementary Table S2) suggests that the strain lacks the signal transduction pathways necessary for light quality dependent regulation of PBS composition^14^. The genes included in the homology search involved *ccaS* (present in CA2 but also in CA7), *pecA* (CA7), *rcaE* (CA3) and *rfpA* (CA6). Since none of these genes was found in the *Cyanobium* genome (Supplementary Table S2), and we did not identify Chl *d* or Chl *f* typical for CA6 or phycourobilin typical for CA4 in *Cyanobium* cells, we conclude that the strain lacks all known CA pathways and is not a chromatic acclimator.

### *Cyanobium* grows with the highest rate under near far-red light

The maximum specific growth rate (µ_max_) of *Cyanobium*, derived from the Gompertz equation^39^, was generally low. It was ranging between 0.37 ± 0.03 and 0.91 ± 0.06 week^-1^ (0.05 ± 0.00 and 0.13 ± 0.01 day^-1^, respectively) which was approx. 2–6 times slower compared to *Cyanobium gracile*^27^ and *Synechocystis* sp. PCC 6803^28^. Such slow growth was likely related to the low PSI content in *Cyanobium* (see below), as all attributes of the cultivation setup including light conditions, cultivation medium, atmosphere, or mixing were identical here as in both previous studies.

**Fig. 1:**
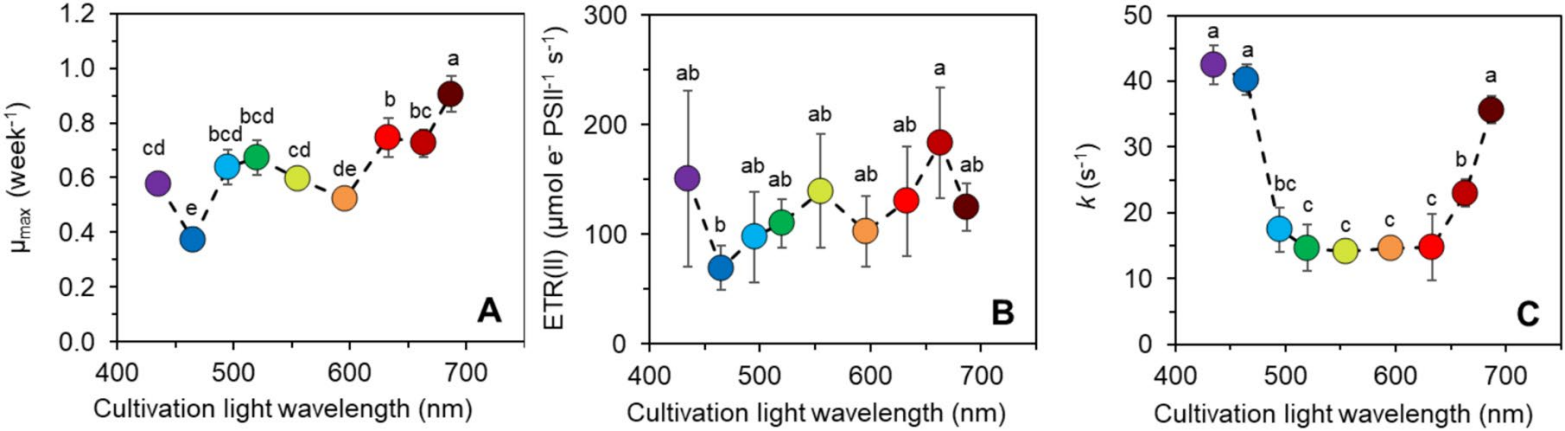
Maximum specific growth rates (**A**), PSII-mediated electron transport rates ETR(II) (**B**), and rates of P_700_^+^ re-reduction kinetics (**C**), as measured after saturation pulse (635 nm, 100 ms; see Materials and Methods for details) in *Cyanobium* cultivated under narrow-band LEDs (Fig. 2). The values represent mean ± SD (n = 3-4). The letters above the symbols indicate statistically significant differences within each parameter (*p* < 0.05).

**Fig. 2:**
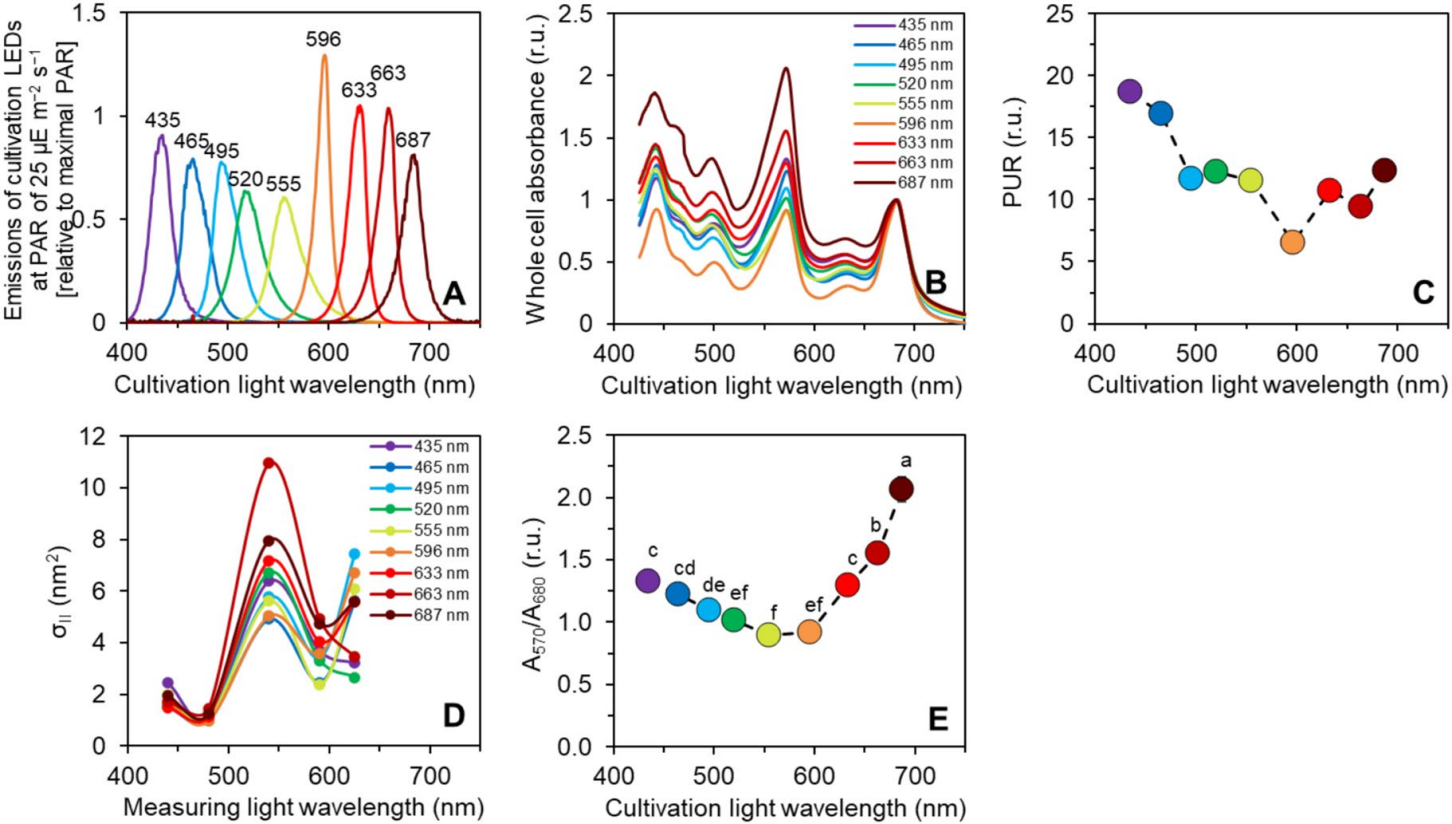
Spectra of cultivation LEDs (**A**), absorption spectra of *Cyanobium* cells (**B**), and resulting values of photosynthetically usable radiation (PUR; **C**), functional absorption cross-section of PSII (σ_II_, **D**) and a ratio of absorbance at 570 nm and 680 nm, calculated from the whole cell absorption spectra (**E**), measured in *Cyanobium sp*. cells cultivated under monochromatic lights. The absorbance spectra were baseline corrected and normalized to the 680 nm Chl *a* Q-band. Values in panels B-E represent averages from 3–4 biological replicates, error bars in panels B-D are omitted for clarity. The letters above the symbols in panel E indicate statistically significant differences within the A_570_/A_680_ parameter (*p*< 0.05).

The growth rate of *Cyanobium* was wavelength dependent: similar to other cyanobacteria, it was the slowest under blue light (465 nm)^27,28,48^. On the contrary, *Cyanobium* was growing the fastest under near far-red light (687 nm; Fig. 1A). The slow growth under blue light was not caused by low PUR (Eq. 11, Fig. 2A–C). It was rather related to the lowest functional absorption cross-section of PSII (σ_II_; Fig. 2D), leading to low PSII-mediated electron transport rate, ETR(II) (Fig. 1B). Since PSI-mediated electron flow (quantified as the rate constant of the P_700_^+^ re-reduction kinetics) under the blue LED was high (Fig. 1C), *Cyanobium* faced strong imbalance between electron flows through PSII and PSI, presumably leading to an imbalance in ATP and NADPH formation under 465 nm light^49^.

The specific growth rate was further low under violet (435 nm) and orange (596 nm) lights. Under violet light, PUR was the highest from all tested wavelengths (Fig. 2C). However, since phycobilisomes absorb violet photons better than blue photons^50^, more energy could be transferred to PSII under 435 nm than under 465 nm, providing space for light harvesting optimization and ETR(II) increase (Fig. 1B). Nevertheless, the highest PUR values under both violet and blue lights were not translated to biomass production (specific growth rate) which was the highest under near far-red lights (Fig. 1A). Since violet and blue light is absorbed by Chl much better than by PBS, the reduced growth rate under both 435 nm and 465 nm LEDs highlights the optimality of light harvesting via PBS, compared with internal Chl antenna of photosystems—in line with previous works^51^.

Under orange cultivation light (596 nm), the reduced specific growth rate (Fig. 1A) was likely related to the lowest measured PUR values (Fig. 2C). The low PUR originated in the combination of the narrow-band emission spectrum of the orange LED (peak at 596 nm, half-width 10 nm) and the weak absorption of phycobilisomes around 600 nm (Fig. 2A-B). Specifically, the absorption spectra of PE, PC, and APC exhibit a pronounced gap in this spectral region^50^.

Interestingly, the highest PUR values were not measured under green (520–555 nm) or red lights (633–663 nm), where either PE or PC exhibit the absorption maxima, respectively. Even though the PUR values were slightly increased in these spectral regions (along with specific growth rates; Fig. 1A), the main effect of PE and PC absorption was pronounced in σ_II_ (Fig. 2D).

**Fig. 3:**
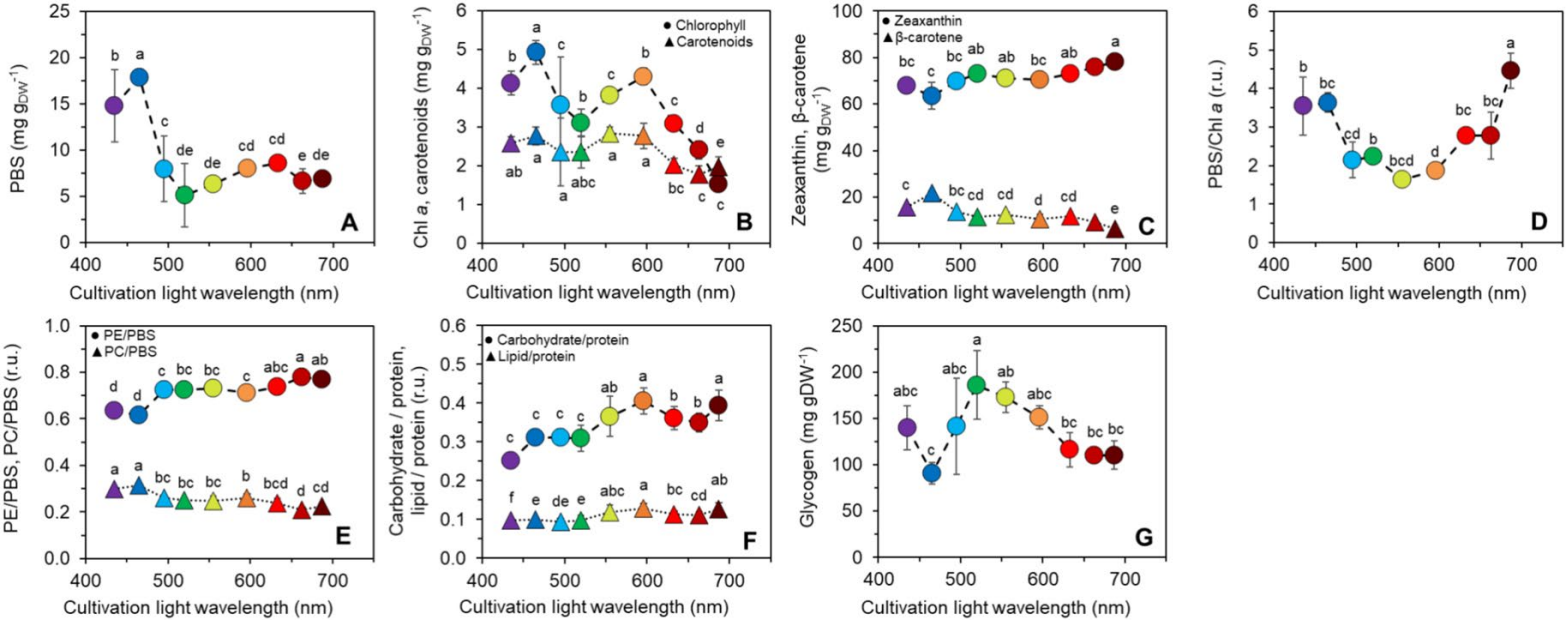
Macromolecular composition of *Cyanobium* cells under narrow-band cultivation lights. Cellular content of phycobiliproteins (PBS) as a sum of phycoerythrin, phycocyanin and allophycocyanin (**A**), content of Chl *a* and total carotenoids (**B**), content of zeaxanthin and β-carotene (**C**), the ratio of phycobiliproteins relative to Chl *a*, based on biochemical assays (**D**), relative content of phycoerythrin, PE, and phycocyanin, PC, in *Cyanobium* phycobilisomes (**E**), content of carbohydrates and lipids in *Cyanobium* cells, relative to protein content (**F**) and cellular content of glycogen (**G**). The values represent mean ± SD (n = 3–4), the letters above the symbols indicate statistically significant differences within each parameter (*p*< 0.05).

**Fig. 4:**
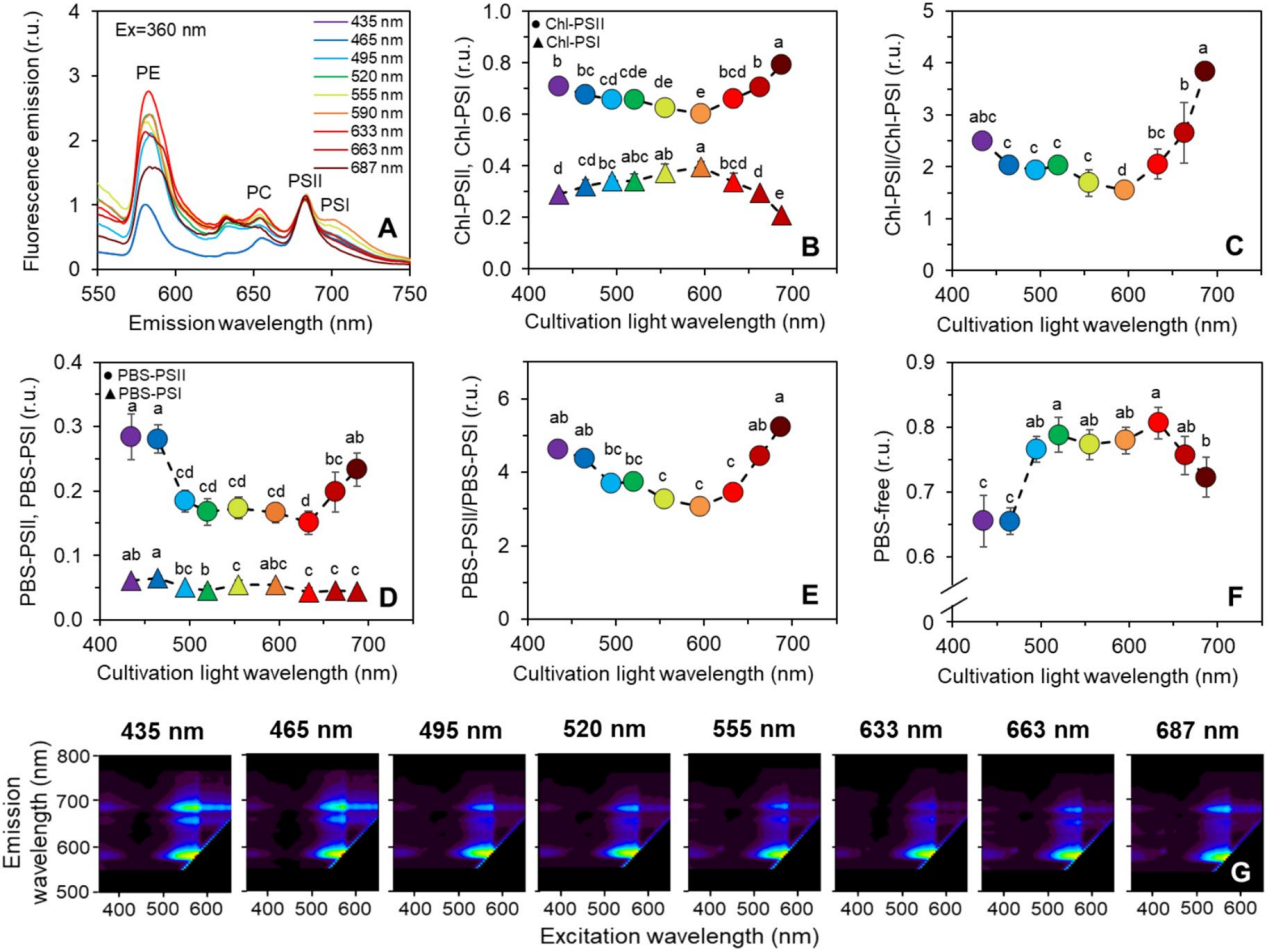
Low temperature (77 K) fluorescence emission spectra of *Cyanobium* upon excitation at 360 nm (**A**), fluorescence emission of Chl *a* originating in PSII and PSI (Eq. 12–13; **B**) and ratio of Chl-PSII/Chl-PSI (**C**), fluorescence emission of phycobilisomes originating in PSII (PBS-PSII) and PSI (PBS-PSI; Eq. 14–15; **D**) and ratio of PBS-PSII/PBS-PSI (**E**), phycobilisomes functionally uncoupled from both PSII and PSI (PBS-free; Eq. 16; **F**) and 3D excitation-emission maps (**G**) of *Cyanobium* cultivated under narrow-band LEDs. The fluorescence spectra in panel A are normalized to emission at 680 nm. The 3D excitation-emission maps in panel G show one representative figure under each cultivation light. The values represent mean ± SD (n = 4–6), the letters above the symbols indicate statistically significant differences within each parameter (*p *< 0.05). Spectra in panel A are presented without error bars for clarity.

The highest growth rate was measured under the near far-red LED (687 nm; Fig. 1A), which allowed for high electron flux through PSI (Fig. 1C). Near far-red light is absorbed efficiently by the internal Chl *a* antennas of both photosystems through their Q-band absorption. In addition, the long-wavelength tails of PC (λ_max_ ≈ 625 nm) and APC (λ_max_ ≈ 650–665 nm) absorption bands extend into the near far-red region (Fig. 2B), providing a secondary but functionally significant pathway for PSII excitation via PBS. The dual excitation of PSI (directly via Chl a) and PSII (via PBS-mediated energy transfer) under 687 nm light thus allows both photosystems to operate simultaneously, unlike shorter wavelengths where excitation is biased toward one photosystem. PSI content in *Cyanobium* decreased dramatically under 687 nm light (Fig. 4) whereas PBS content remained similar compared to red light (Fig. 3). This allowed *Cyanobium* cells to increase PBS coupling to PSII (Fig. 4), securing efficient light harvesting and efficient transfer of the harvested light into PSET (Fig. 1B,C). The wavelength-dependent trends in specific growth rate were not reflected in cell volume, which was independent of the cultivation wavelength (Supplementary Fig. S3).

### The interplay between PSII- and PSI-mediated electron flow rates

The different trends in wavelength dependency of specific growth rate and electron flows through PSII and PSI (Fig. 1) suggest that the relationship between ETR and growth rates is not linear. Indeed, the PSI-mediated electron flow (quantified as the rate constant, *k*, of the P_700_^+^ re-reduction kinetics; Fig. 1C), reflects a sum of linear electron flow (LEF) from PSII, cyclic electron flow through PSI (PSI-CEF) and respiratory electron flow (REF). Further measurement would thus be required to dissect the effect of these individual electron flows on the overall *k* value^28^.

Nevertheless, the highest electron flow through PSI, measured under violet, blue and near far-red lights, corresponds well with several factors. The *k* value integrates the electron flow through all photosystems I in *Cyanobium* cells, whereas ETR(II) is expressed as the flow of electrons per PSII. Under 435 nm and 465 nm cultivation lights, PUR (Fig. 2C), total PBS amount in *Cyanobium* cells (Fig. 3A) as well as PBS attachment to both PSII (PBS-PSII) and to PSI (PBS-PSI) were high (Fig. 4). Nevertheless, even these acclimations did not allow *Cyanobium* to capture blue photons efficiently to avoid growth limitation. As the total LEF was supposedly limited under blue light compared to other wavelengths — given the low σ_II_ and ETR(II) — the high *k* values (Fig. 1C) may reflect elevated PSI-CEF activity rather than REF, which typically increases with increasing growth rate^26^. However, since the *k* parameter integrates all electron donation pathways to PSI, the relative contributions of LEF, PSI-CEF and REF would require additional data to be fully resolved.

Under 687 nm cultivation light, PUR was not as high as under violet and blue lights (Fig. 2C). However, the PSII amount was upregulated, opposite to PSI which was downregulated. At the same time, PBS-PSII was high (Fig. 4). Therefore, the total electron flow through PSII under near far-red light was likely high (also due to higher σ_II_; Fig. 2D), which resulted in a high *k* value. Therefore, the high electron flow rates through PSI under 687 nm cultivation light likely originated from different relative contributions of LEF and PSI-CEF compared to 435 nm and 465 nm lights. Specifically, the upregulated PSII content, high PBS-PSII, and elevated σ_II_ under 687 nm suggest a larger LEF contribution to the overall *k* value, whereas under blue light — where PSII-mediated electron transport is limited — PSI-CEF may predominate. However, direct quantification of these individual electron flows would be needed to confirm this interpretation.

Interestingly, the *k* values were low under 495–633 nm cultivation lights (Fig. 1C). In this yellow/green wavelength region, PE absorbs well—as reflected in absorbance spectra and high σ_II_ (Fig. 2)—which allows for high ETR(II) (Fig. 1B). However, PBS content (Fig. 3A), PSII content (Fig. 4B), and PBS-PSII were low (Fig. 4), limiting the LEF contribution to the overall *k* value.

### Light capture characteristics and implications for PUR and σ_II_

Cell absorption spectra show peaks typical for PE (570 nm), PC (625 nm), Chl *a* (440 and 680 nm) and carotenoids (495 nm; Fig. 2B)—pigments detected by biochemical assays (Fig. 3). Interestingly, even though the highest absorption peaks were measured around 570 nm and 680 nm, the highest PUR values were found under 435 nm and 465 nm LEDs, due to generally efficient *Cyanobium* absorption in the violet/blue part of the light spectrum. On the contrary, σ_II_, representing the effective area through which PSII absorbs light energy driving photosynthesis^32^, was the highest at 540 nm and 625 nm (of the measuring light of the fluorometer; Fig. 2D, Supplementary Figure S4). σ_II_ is derived from Chl fluorescence measurements. Its value thus reflects not only a sole light absorption, but rather an efficient light capture and transfer to PSII and to PSET under a given wavelength. High σ_II_ was the main reason for high ETR(II) measured under yellow/green and red cultivation lights (Fig. 1B). However, as discussed above, due to other factors such as shifts in PSII, PSI and total PBS content, as well as in PBS-PSII and PBS-PSI (Figs. 3, 4), the translation of σ_II_ values to the growth rates of *Cyanobium* was only weak (Fig. 1A).

Interestingly, the lowest σ_II_ values were measured under 440 nm and 425 nm (of the measuring light of the fluorometer). This is in high contrast with high absorption of *Cyanobium* cells and high PUR values under 435–465 nm cultivation lights (Fig. 2A–C). This shows a weak transfer of the captured light energy to PSII-mediated PSET in this part of the light spectrum—confirming the importance of PBS in light harvesting in cyanobacteria. Overall, the absolute σ_II_ values correspond with previously reported values in PC-rich strains *Cyanobium gracile* ^27^ and *Synechocystis* sp. PCC 6803^28^.

### Shifts in cell composition suggest wavelength-dependent metabolic reorganization

From the absorption spectrum of *Cyanobium* cells (A_570_/A_680_), it was possible to estimate the ratio of PE/Chl *a*. This parameter, showing minimal PE concentration under yellow/orange cultivation light, and maximum under 687 nm cultivation light (Fig. 2E), corresponded well with the PE/Chl *a* concentrations estimated by biochemical assays (Supplementary Fig. S5; Fig. 3D). Since the total PBS amount was the highest under violet/blue cultivation lights (Fig. 3A), the relatively high PE content in *Cyanobium* cells under near far-red light was rather related to PE increase within PBS (Fig. 3E) and high PBS/Chl *a* ratio (Fig. 3D) due to low PSI content (Fig. 4B). The PE shift within PBS was not related to chromatic acclimation. Instead, *Cyanobium* appears to represent a PE-rich spectral generalist. The presence of the *cpcL* gene suggests that, analogous to the situation described in *Anabaena* sp. PCC 7120^52^, *Cyanobium* likely harbors two distinct PBS morphologies — *CpcL*-associated rod-shaped PBS and conventional hemidiscoidal PBS, both enriched in PE-containing discs.

Besides shifts in PBS composition, also levels of Chl *a* and carotenoids were dependent on the cultivation wavelength. Content of the only identified chlorophyll, Chl *a*, was the lowest under 687 nm, same as content of total carotenoids (Fig. 3B). Since PSI contains about 3-times more Chl *a* than PSII^53^ this Chl *a* shift is consistent with low PSI amount measured under near far-red lights (Fig. 4B). This result is further consistent with shifts in individual carotenoids: β-carotene can be expected to follow PSII and PSI content as structural carotenoid^53,54^, whereas xanthophylls are typically upregulated under the conditions of high photosynthetic activity, or generally under stress conditions^28,48^. The relatively high zeaxanthin content under 687 nm cultivation light thus corresponds well with maximal growth rates under this wavelength (Fig. 1A). The content of carotenoids, relative to Chl *a*, was higher than previously reported for *Cyanobium gracile*^27^ and *Synechocystis* sp. PCC 6803^28^.

Color of the cultivation light also affected macromolecular cell composition. The content of carbohydrate, relative to protein, was generally higher under orange/red light, compared to violet/blue/green light. A similar trend was observed for relative content of lipids (Fig. 3F). Interestingly, the amount of glycogen was the most upregulated under yellow/green lights (Fig. 3G). Glycogen is built from glucose, with only ATP but no NAD(P)H required for its synthesis, and it works as an energy buffer in cyanobacteria^55^. Notably, glycogen accumulation was highest under yellow/green light (Fig. 3G), where PBS-mediated light harvesting was efficient (high σ_II_,Fig. 2D) but electron flow through PSI was low (Fig. 1C). In contrast, glycogen levels were lower under violet/blue and near far-red lights, where electron flow through PSI was high (Fig. 1C) but growth rates varied considerably (Fig. 1A). The observation that glycogen accumulation did not follow the same wavelength dependency as either growth rate or carbohydrate content suggests that the partitioning of fixed carbon between glycogen and other cellular components is regulated differently across the light spectrum. These divergent trends are consistent with wavelength-dependent differences in the balance of photosynthetic energy carriers, although direct quantification of ATP and NADPH levels would be required to test this hypothesis. Overall, the macromolecular composition data indicate rather complex metabolic regulation across the tested light spectrum, which is only partially related to efficient light absorption by PE and increased σ_II_ under yellow/green light.

### PBS attachment to PSII and PSI, and PSII/PSI ratio fine-tune light harvesting

*Cyanobium* had generally low content of PSI, relative to PSII—as estimated from low temperature (77 K) fluorescence emission spectra (Fig. 4A). The PSI content was similarly low as in close relative strain *Cyanobium gracile*^27^ but lower than in other cyanobacteria, such as *Synechocystis* sp. PCC 6803^28^. Interestingly, Chl-PSII and Chl-PSI parameters (Eqs. 12–13), representing abundance of individual PSII and PSI on a semi-quantitative basis, showed opposite trends of dependency on the cultivation wavelength (Fig. 4A). This suggests that with shifting cultivation wavelength, PSII was upregulated simultaneously with PSI downregulation, and vice versa. The resulting PSII/PSI ratio was the highest under 687 nm cultivation light (Fig. 4C)—which, together with the highest ratio of PBS-PSII/PBS-PSI (Fig. 4E), helped to optimize light absorption under near far-red light, leading to the highest specific growth rate among all tested spectral regions (Fig. 1A).

Interestingly, in *Cyanobium* cells cultivated under white light of PFD 25 μmol photons m^−2^ s^−1^, fluorescence emission from PSI was slightly higher compared to emission from PSII, suggesting lower PSII/PSI ratio compared to the ratio obtained under individual narrow-band LEDs (Supplementary Figure S6). This demonstrates an additional layer of complexity in photosynthetic and likely also metabolic regulation under white light, where multiple wavelengths are combined. Additionally, the PSII/PSI ratio shifts under increasing light intensity (Supplementary Figure S6). However, since this study focuses solely on light quality, the effect of light intensity was not investigated further.

Besides PSII and PSI content, PSET efficiency was fine-tuned via PBS attachment to PSII or PSI. The highest values of PBS-PSII were measured under violet, blue and near far-red lights (Fig. 4D-E). As discussed above, PBS attachment to PSII contributed to the relatively high growth rate under 687 nm and, to a lesser extent, under 435 nm cultivation lights (Fig. 1A). Under 465 nm blue light, however, the equally high PBS-PSII did not translate into improved growth. This is likely because the functional benefit of PBS-PSII depends not only on the degree of PBS attachment but also on the ability of PBS to absorb photons at the given wavelength. Since PBS absorbs violet photons (435 nm) more efficiently than blue photons (465 nm)^50^, the high PBS-PSII under blue light did not compensate for the intrinsically low light capture by PBS in this spectral region, leaving the PSII excitation rate — and consequently ETR(II) (Fig. 1B) — insufficient to support fast growth. Under 687 nm, where both the Chl *a* Q-band and the long-wavelength tails of PC and APC absorption contribute to light harvesting (Fig. 2A-B), the high PBS-PSII could effectively channel absorbed energy into PSET. Interestingly, the highest proportion of PBS was found functionally detached from both photosystems (Eq. 16,Fig. 4F). The relative content of PBS-free was higher compared to *Synechocystis* sp. PCC 6803^28^. This highlights generally inefficient light harvesting in *Cyanobium*, which was likely related to generally slow specific growth rates, as discussed above.

### Redox state of the PQ pool varied with cultivation wavelength but remained more oxidized than in related strains

**Fig. 5:**
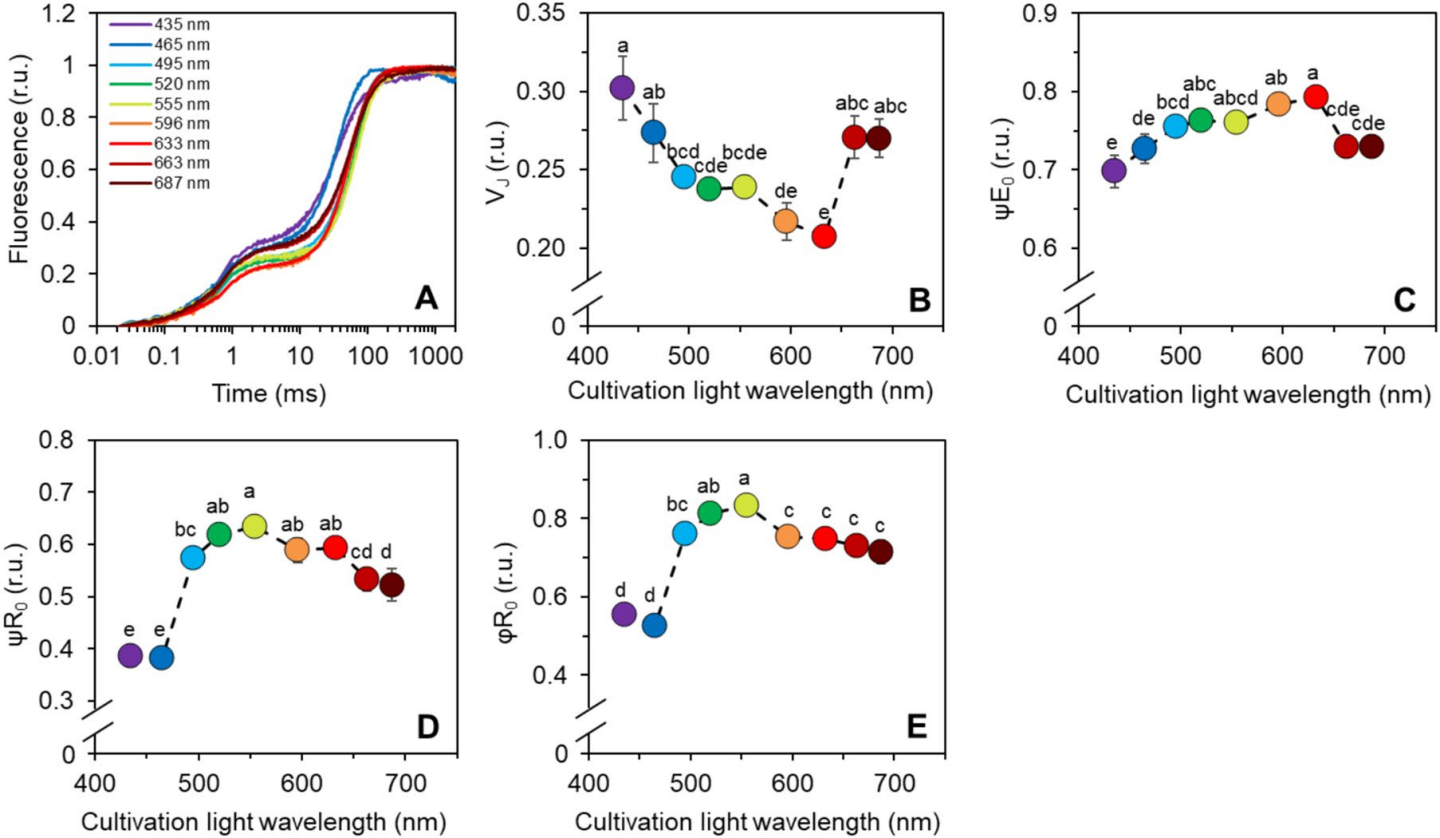
Fast Chl *a* fluorescence induction curves (OJIP curves; **A**) measured in *Cyanobium* cultures cultivated under narrow band LEDs. The derived parameters represent proxy of the redox state of plastoquinone pool (V_J_; **B**), efficiency with which a PSII-trapped electron is transferred to PQ (ψE_0_; **C**) or to PSI (ψR_0_; **D**), and overall quantum yield of electron transport from PSII to PSI (φR_0_; **E**). The values represent mean ± SD (n = 3–4), the letters above the symbols indicate statistically significant differences within each parameter (*p*< 0.05). Spectra in panel A are between F_O_ and F_M_ (initial and maximal fluorescence, respectively) and are presented without error bars for clarity.

Further insight into the photosynthetic efficiency can be obtained from the analysis of fast Chl *a* fluorescence induction curves (Fig. 5A). The parameter V_J_ (Eq. 6) showed the highest values under violet, blue and near far-red lights (Fig. 5B), suggesting a reduced PQ pool under these cultivation lights compared to other wavelengths^34,35^. This could have several origins: since electron flow through PSI under these wavelengths was high (Fig. 1C) and ETR(II) did not shift (Fig. 1B), the higher V_J_ values suggest either high REF potentially pumping electrons from metabolism to the PQ, or limitation of PSI on the acceptor side, potentially imposing a block for PQH_2_-trapped electrons. The higher V_J_ values under these wavelengths can be also related to higher PBS-PSII and lower PBS-free content (Fig. 4D-E). The parameter V_J_ was generally lower than in *Synechocystis* sp. PCC 6803 as well as in *Cyanobium gracile* cultivated under identical cultivation conditions^27,28^. Thus, while the PQ pool in *Cyanobium* sp. NIVA-CYA 375 showed clear wavelength-dependent redox modulation — being more reduced under violet, blue and near far-red lights — its overall redox state remained more oxidized than in the comparison strains, consistent with the generally lower photosynthetic electron transport rates in this organism.

The parameters ψE_0_, ψR_0_ and φR_0_, describing the efficiencies of transfer of a PSII-trapped electron to PQ and to PSI, and the overall quantum yield of electron transport from PSII to PSI, respectively, were all downregulated under violet and blue cultivation lights (Fig. 5C-E), further confirming inefficient light harvesting under these wavelengths.

### State transitions fine-tune light harvesting and NPQ

**Fig. 6:**
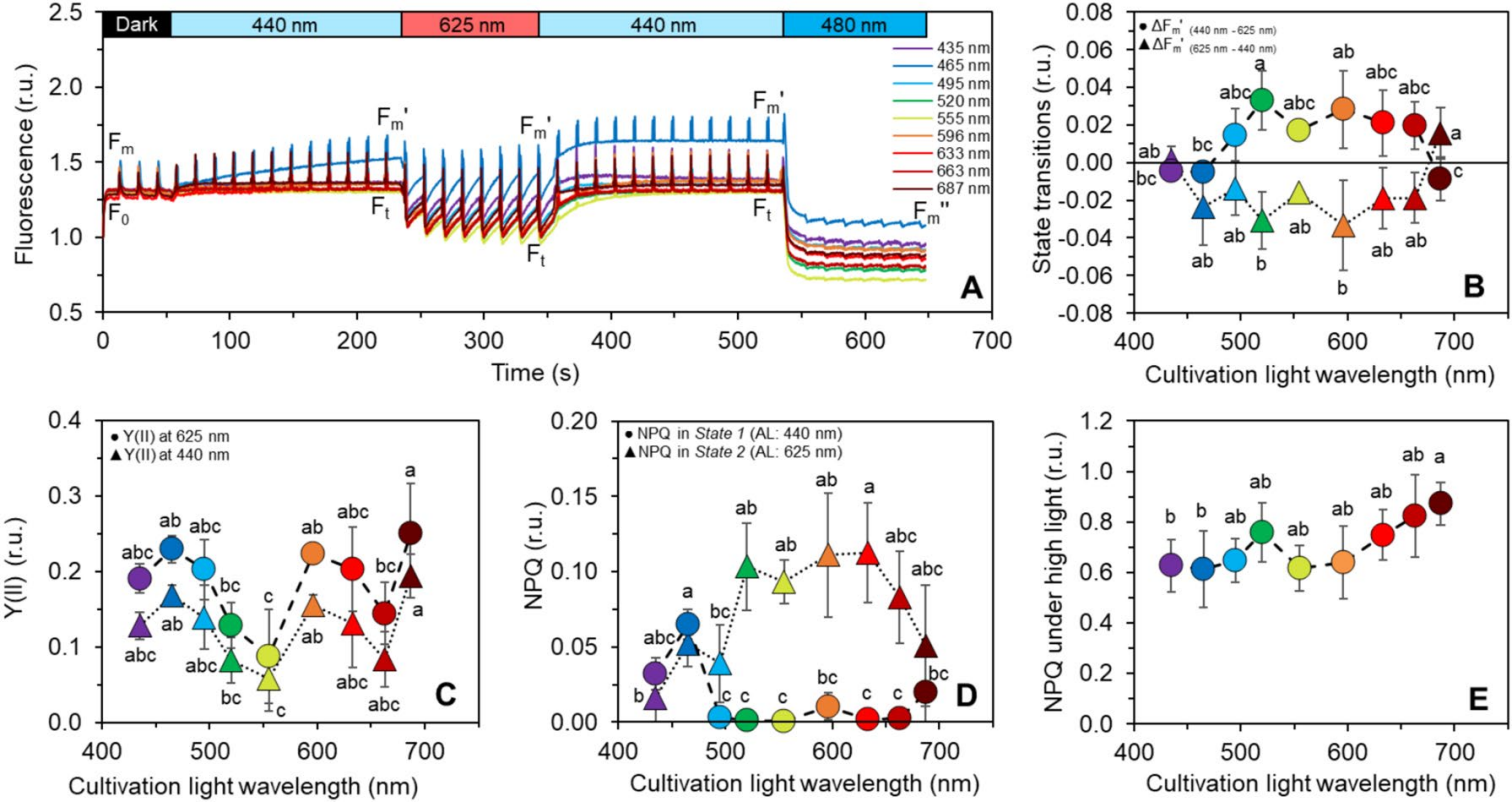
Chlorophyll *a* fluorescence kinetics in *Cyanobium* cultures cultivated under narrow-band LEDs upon illumination by 440 nm and 625 nm actinic light (AL) of intensity 100 μmol photons m^−2^ s^−1^ and by 480 nm light of intensity 1 500 μmol photons m^−2^ s^−1^ (**A**). From the chlorophyll *a* fluorescence kinetics, state transitions (**B**), effective quantum yield of PSII (**C**) and non-photochemical quenching was estimated (**D**). The intensity of *State 1* → *State 2* transition was estimated from F_m_’ shift upon illumination of blue-light acclimated cultures by 625 nm AL. Reciprocally, *State 2* → *State 1* transition was estimated from F_m_’ shift upon illumination of red-light acclimated cultures by 440 nm AL. The values represent mean ± SD (n = 3–4), the letters above the symbols indicate statistically significant differences within each parameter (*p*< 0.05). Spectra in panel A, normalized to initial fluorescence level, are presented without error bars for clarity.

Measurement of slow Chl *a* fluorescence transients under 440 nm and 625 nm AL (Fig. 6A), inducing *State 1* and *State 2*, respectively^20^), revealed that state transitions (ST) in *Cyanobium* were dependent on the cultivation light wavelength. Cultures grown under 435 nm and 465 nm LEDs exhibited the weakest ST (Fig. 6B)—which was likely related to the overall high PBS (Fig. 3A) and PBS-PSII, and low PBS-free content (Fig. 4). In contrast, higher ST amplitudes in the 495–663 nm range suggest that *Cyanobium* attempted to further optimize light harvesting in this wavelength range. Interestingly, under 687 nm cultivation light, ST showed the opposite pattern compared with all other wavelengths, further distinguishing this illumination from the rest of the cultivation lights. The ST intensity was lower compared to *Synechocystis* sp. PCC 6803 and *Cyanobium gracile*, which was again likely related to the overall higher photosynthetic capacity in those strains compared with *Cyanobium* sp. NIVA-CYA 375^27,28^.

In *State 2* induced by 625 nm AL, *Cyanobium* showed higher efficient quantum yield of PSII, Y(II) (Eq. 3; Fig. 6C). Since the *State 2* is characterized by decreased σ_II_ and increased σ_I_ (absorption cross-section of PSI;^20^), the higher Y(II) in *State 2* is likely a result of reduced light absorption by PSII, with more absorbed light being used in PSET. The Y(II) trend across the cultivation light spectrum—being the lowest under 555 nm and 633 nm cultivation lights—correspond well with overall Chl *a* and PBS content in *Cyanobium* cells (Fig. 3A-B).

State transitions also affected NPQ, which showed opposite trends in *State 1* and *State 2*, when induced by AL of moderate light of 100 μmol photons m^−2^ s^−1^ (Fig. 6D). Under 1 500 μmol photons m^−2^ s^−1^ of 480 nm AL, NPQ was significantly higher, and the most pronounced under 435 nm, 465 nm and 687 nm cultivation lights (Fig. 6D), corresponding well with high PBS-PSII content (Fig. 4D) and PBS/Chl *a* ratio (Fig. 3D).

Interestingly, the steady-state fluorescence (F_t_) decreased after SP applied under 625 nm AL (Fig. 6A), opposite to 440 nm AL. This drop, previously observed in *Synechocystis* sp. PCC 6803 under the light conditions where PSI content was reduced^28^, was assigned to a temporal limitation of linear electron flow on the PSII acceptor site^56^. These results correspond well with generally low PSI content in *Cyanobium* cells measured here (Fig. 4).

## Discussion

The photosynthetic apparatus of cyanobacteria is highly plastic, allowing these organisms to thrive in diverse and fluctuating environments. This plasticity is achieved through mechanisms ranging from long-term adjustments in pigment composition and photosystem stoichiometry to short-term regulation of electron flow and state transitions, and is key for ecological success and persistence of these organisms across a broad range of habitats^20,57^. Among all environmental factors driving cyanobacterial fitness, light quality—i.e., the spectrum of light available—was for a long time the least studied. However, as recognized recently, light quality is one of the key factors driving abundance of phytoplankton on a global scale^12^. This is due to the specifics of underwater attenuation of the sunlight spectrum. Vibrational modes of water molecules absorb photons at specific wavelengths, providing the basis for so-called spectral niches within the underwater spectrum^13^. On top of the ecological relevance, light quality has been recognized as a factor increasing phototrophic production of bulk chemicals^48^.

In this study, we characterized spectral acclimation of *Cyanobium* sp. NIVA-CYA 375, a cyanobacterium lacking the ability to perform chromatic acclimation (see next paragraph for further details). By comparing the response of this strain to narrow-band spectra within the entire PAR wavelength range, we identify NIVA-CYA 375 as a strain with distinct strategies for utilizing near far-red light and mitigating blue light stress compared to previously studied *Synechocystis* sp. PCC 6803 and the phylogenetically related *Cyanobium gracile* (a CA1 strain and a non-CA strain, respectively, both rich in PC content). This comparative characterization across the full PAR spectrum provides details of light acclimation strategies on top of the chromatic acclimation mechanism.

Despite genomic analysis of *Cyanobium* sp. NIVA-CYA 375 revealing the presence of *cpeA* and *cpcL* genes, the strain is not a chromatic acclimator. Instead of possessing the complete set of CA regulatory genes, *Cyanobium* cells exhibit two distinct PBS morphologies that may vary depending on the wavelength of the growth light. Although *Cyanobium* modulates PE content in response to light quality, the presence of the *CpcL* linker allows preferential attachment of PBS to PSI under specific conditions, helping to balance excitation pressure^15^. This organization resembles that of *Cyanobium gracile*, which also lacks CA capability^14^, as well as *Synechocystis* sp. PCC 6803, where acclimation to light quality involves remodeling of PBS rod composition rather than synthesis of phycobiliproteins. Despite lacking CA capability, *Cyanobium* sp. NIVA-CYA 375 maintains a broad pigment repertoire — including both PE and PC, on top of Chl *a* — that enables light absorption across most of the PAR spectrum. In this sense, *Cyanobium* acts as a spectral generalist in terms of its light-harvesting capacity, in contrast to *Cyanobium gracile* or *Synechocystis* sp. PCC 6803 that rely predominantly on PC. However, our data show that this broad absorption capacity does not translate into uniformly efficient growth across all wavelengths. Rather, our data show that efficient growth is achieved only under wavelengths that excite both photosystems simultaneously, suggesting that broad spectral absorption is a necessary but not sufficient condition for optimal growth. Nevertheless, the ability to harvest light across the spectrum may still provide a competitive advantage in the spectrally variable light fields of turbid, shallow lakes, where no single wavelength dominates persistently. It should be noted, however, that reconstruction of natural underwater spectra is more complex than the narrow-band conditions applied here, and would require light sources composed of multiple wavelengths^13,58^. Nevertheless, the narrow-band cultivation LEDs cover the absorption peaks of all major light harvesting pigments in *Cyanobium*, including Chl *a* (435 nm and 687 nm), PE (555 nm), PC and APC (633 nm) and also carotenoids (495 nm, Fig. 2A). As such, they provide a useful framework for identifying potential PSET limitations across the PAR spectrum and allow estimation of the potential fitness of *Cyanobium* even within more complex spectral environments.

Environmental conditions under which such a competitive advantage can be expected include turbid or eutrophic waters where the underwater light spectrum contains a substantial fraction of far-red photons^13,58^. Red and near far-red light are also enriched in the incident solar spectrum during winter months, when the lower solar elevation angle increases the optical path length through the atmosphere. This enhances Rayleigh scattering of shorter wavelengths (blue/violet), and thereby shifts the spectral balance toward longer wavelengths^59^.

Depending on the cultivation wavelength, differences in the PE content of PBS were observed. However, PE levels were higher under red light than under green light (Fig. 3E), opposite to the pattern reported for the CA2 strain *Nostoc punctiforme* PCC 73102^60^. Similarly, the increased attachment of PBS to PSI under yellow-green light, previously described in *Leptolyngbya* sp. PCC 6406^14^ was only partially observed here (Fig. 4D-E), whereas more pronounced wavelength-dependent differences occurred in PBS–PSII association and the PSII/PSI ratio (Fig. 4B-E). Together, these observations suggest responses similar to CA, however, in *Cyanobium* the underlying mechanism appears to rely on structural diversity of PBS rather than on canonical CA regulatory pathways.

*Cyanobium* sp. NIVA-CYA 375 exhibited exceptionally low PSI content relative to PSII (Fig. 4). This feature was not an acclimatory response to specific wavelengths but rather an inherent characteristic of the strain. Even under optimal growth conditions (687 nm), PSI content remained low and in fact decreased most strongly among all tested wavelengths (Fig. 4). Such low PSI content imposes an intrinsic acceptor-side limitation for electrons originating in PSII, as indicated by a dip in steady-state fluorescence after SP application (Fig. 6A)^27,28^.

Total electron flow through PSI was the highest under violet/blue and near far-red illumination, all of which excite Chl *a*—and thus PSI—efficiently (Fig. 1C). However, the biological consequences of high electron flow through PSI likely differed between these spectral regions. Under violet/blue light, where PSII-mediated electron transport is constrained (Fig. 1B), the high *k* values may largely reflect elevated PSI-CEF, potentially leading to over-production of ATP relative to NADPH. In contrast, under 687 nm light, the concurrent upregulation of PSII content and PBS-PSII (Fig. 4) suggests that a substantial fraction of the PSI electron flow originated from LEF, thereby maintaining a more balanced NADPH/ATP output. While direct dissection of LEF and PSI-CEF contributions was beyond the scope of this study, this interpretation is consistent with the observation that 687 nm supported the highest growth rate whereas violet/blue light did not (Fig. 1A), despite similarly high *k* values. NADPH and ATP formation is typically tightly regulated in cyanobacteria^61,62^. However, NADPH/ATP imbalance, together with insufficient NADPH production, has previously been identified as a key growth-limiting factor under blue light in PC-rich strain *Synechocystis* sp. PCC 6803^22,49,63^. Consistently, and in agreement with previous studies^27,28,64^ *Cyanobium* exhibited its lowest specific growth rate under blue light. Under violet/blue and near far-red illumination, the *Cyanobium* also reduced PSI content most strongly, suggesting an attempt to compensate for imbalanced excitation of PSII and PSI.

In contrast, *Cyanobium* sp. NIVA-CYA 375 exhibited its highest growth rates under near far-red light (Fig. 1). This response contrasts sharply with that of *Synechocystis* sp. PCC 6803 and *Cyanobium gracile*, both of which show markedly reduced growth under this wavelength range^27,28,65^. In *Synechocystis*, impaired growth under near far-red light has been linked to strong upregulation of stress-related genes, including those encoding heat-shock proteins and high-light-inducible proteins^66^. Considering the previously reported high electron flow through PSI under near far-red light in *Synechocystis*^28^, similar to that observed in *Cyanobium* in this study (Fig. 1C), we hypothesize that the elevated growth rates in *Cyanobium* may be related to the absence of such a stress response.

The physiological response in *Cyanobium* to near far-red light differed from its response to blue light. Blue/violet illumination induced upregulation of PBS (Fig. 3A) together with a reduction in the PSII/PSI ratio (Fig. 4C), ultimately decreasing the efficiency of electron transport from PSII to PQ and PSI (Fig. 5). These observations again highlight the importance of PBS-mediated light harvesting. Under violet/blue light, *Cyanobium* attempted to compensate for relatively weak PBS absorption by increasing PBS abundance. In contrast, emission from the 687 nm LED overlaps with the long-wavelength absorption tails of PC and APC (Fig. 2A-B). Although this overlap is not as strong as the absorption at the respective λ_max_ values, it is sufficient to excite PBS and drive energy transfer to PSII, as evidenced by the elevated σ_II_ under this wavelength (Fig. 2D) and the high PBS-PSII coupling (Fig. 4D). This partial but functionally effective PBS absorption under 687 nm is critical, as it enables PSII excitation via PBS concurrently with direct PSI excitation via Chl *a*, thereby balancing the excitation of both photosystems. This balanced excitation, rather than PBS upregulation, appears to be the primary mechanism enabling optimal electron transport and the highest growth rate under near far-red light.

In phytoplankton, optimal light quality for growth generally corresponds to the wavelengths most strongly absorbed by the photosynthetic pigments. Accordingly, green- or blue-light specialists typically grow optimally under green or blue light, respectively^67,68^. PC-rich cyanobacteria usually exhibit optimal growth under orange/red light absorbed efficiently by PC and APC^27,28^, whereas PE-rich strains can be expected to grow best under yellow/green light, consistent with the strong absorption peak and high functional absorption cross-section of PSII in this spectral region (Fig. 2).

In contrast to these expectations, *Cyanobium* grew optimally under 687 nm illumination. Notably, the specific growth rates under green and red light were similar (Fig. 1A), despite the lowest PSII quantum yields occurring under these wavelengths (Fig. 6C). Together with relatively low PBS–PSII and high PBS-free fractions (Fig. 4), this observation indicates that although PBS-mediated light harvesting was efficient, the captured excitation energy was not fully utilized for photosynthetic electron transport. This is consistent with the acceptor-side limitation of PSII described above (Figs. 4,6). Optimal growth under 687 nm illumination therefore appears to result from simultaneous excitation of both PSII via PBS and PSI via Chl *a*. Overall, the light-quality acclimation of *Cyanobium* sp. NIVA-CYA 375 thus illustrates how low PSI content can redirect the spectral optimum for growth toward wavelengths that enable balanced excitation of both photosystems — a constraint that fundamentally shapes the wavelength dependence of cyanobacterial fitness.

## Supplementary Information

Supplementary Information.

## Data Availability

All data and material are available upon request of the corresponding author. Additionally, we provide a freely accessible online tool for streamlined processing and preliminary analysis of fluorescence data from kinetic fluorometers and spectrofluorometers as well as statistical analysis used in this study, available at https://www.cyano.tools/. The generated genomic data have been deposited in the NCBI BioProject database under the accession numbers PRJNA1377438 and SAMN53770833.
