# Supplementary material for: Low PSI content broadens the optimal light spectrum for a phycoerythrin-dominated cyanobacterium towards near far-red
Source: Sci Rep. 2026 May 7;16:21079. doi: 10.1038/s41598-026-50772-z (PMC13342260; doi:10.1038/s41598-026-50772-z)
Supplement: Supplementary file 1 — Supplementary Information. [file 41598_2026_50772_MOESM1_ESM.docx]

**Supplementary information**

**Low PSI Content Broadens the Optimal Light Spectrum for a Phycoerythrin-Dominated Cyanobacterium towards Near Far-Red**

Mariann Kis^1^, Tomáš Zavřel^2^, István Fodor^1^, Anna Segečová^2^, Péter Urbán^4^, Bence Gálik^4^, Róbert Herczeg^4^, Attila W. Kovács^1^, László Kovács^3^, Martin Lukeš^5^ and Gábor Bernát^1^

^1^HUN-REN Balaton Limnological Research Institute, Tihany, 8237, Hungary

^2^Global Change Research Institute, CAS, Brno, 603 00, Czech Republic

^3^HUN-REN Biological Research Centre, Szeged, 6726, Hungary

^4^Genomics and Bioinformatics Core Facilities, Szentágothai Research Centre, University of Pécs, Pécs, 7624, Hungary

^5^Centre Algatech, Institute of Microbiology, 379 01 Třebon, Czech Republic

**Supplementary Table S1: Summary of *de novo* genome assembly statistics.** Assembly metrics (computed using QUAST v5.2.0) for the draft genome generated from contigs obtained after quality filtering. Statistics include the total number of contigs at different length thresholds, total assembly length, largest contig size, N50/N90 values, L50/L90 values, auN, and GC content. Note: N50 - the contig length for which 50% of the total assembly length is contained in contigs of that length or longer; L50 - the minimum number of contigs whose cumulative length accounts for 50% of the total assembly size; N90 and L90 correspond to the 90% cumulative assembly threshold; auN - area under the Nx curve.

*
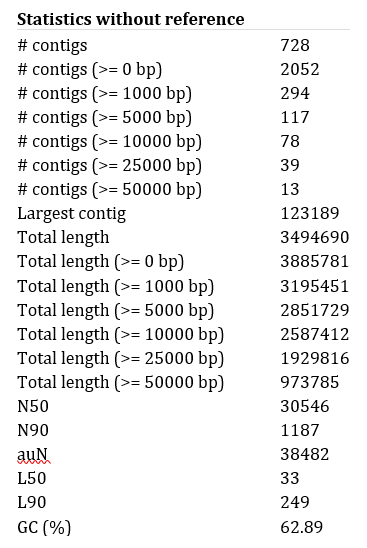
*

**Supplementary Table S2:** Presence of genes associated with chromatic acclimation (CA) types in the genome of *Cyanobium* sp. NIVA-CYA 375. The table lists genes involved in chromatic acclimation types CA0–CA7 and indicates whether each gene was detected (“Hit: Yes/No”) in the genome. CA type assignments and gene functions are based on prior literature [(Hirose et al. 2019; Sanfilippo et al. 2019; Mondal et al. 2024)](https://paperpile.com/c/Fc16UX/VrQ6+VUyf+X6rB). The coding sequence of the identified CpcL and CpeA genes are provided below.


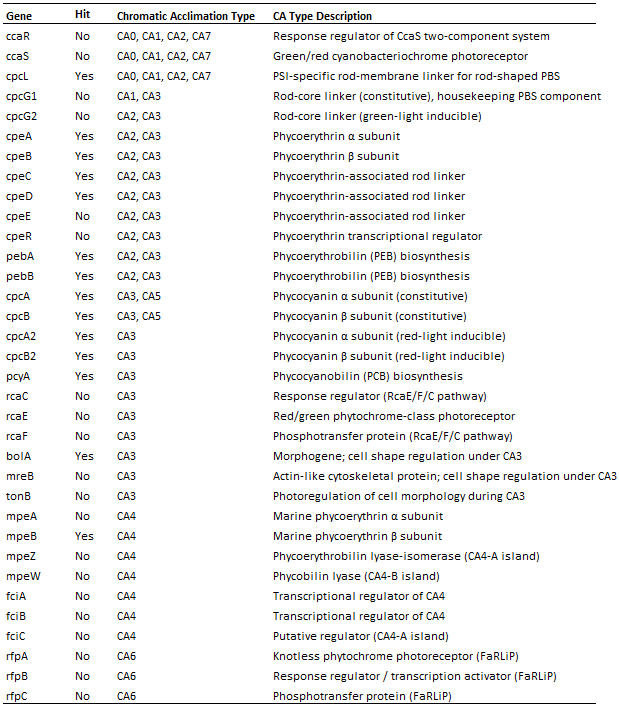


> Cyanobium_NIVA-CYA 375_CpeA

ATGAAGTCTGTTGTGACCACTGTTGTGACCGCCGCTGATGCCGCTGGTCGCTTCCCCTCCCAGAACGACCTAGAAGCTGTTCAGGGTAACATTCAGCGAGCTACTGCTCGTCTTGAAGCTGCTGAGAAGCTGGCTGCCGGCCTGGACAAAGTGACGAAGGAAGCTGGTGACGCCTGCTTCAGCAAGTATCCCTACCTCAAGCAAGCCGGCGAAGCTGGTGAGAATCAGGTCAAGGTGGACAAGTGCTACCGCGACATCGCTCACTATCTGCGTCTGATCAACTACTGCCTGGTTGTGGGCGGCACCGGCCCTCTCGATGAGTGGGGCATTGCCGGAGCCCGTGAGGTGTACCGCACCCTGCGTCTGCCTACCGCTGCTTACGTGGAAGCTCTCACCTTCACCCGCGATCGGGCTTGCTCCCCTCGTGACATGAGCCCCCAGGCTCTCAATGAGTTGAAGTCGTACCTTGATTACGCAATCAACGCCCTCTCC

> Cyanobium_NIVA-CYA 375_CpcL

ATGCCCTTGCCCGTTCTGGCCACCAAGCCACTCACCAACAGCGCTCGCGTCAGCAGCTTTCTGGCCGCCGGTGAGGAAAGCCCCCGCCAGAGCGACACCACTTGCCTGGTACGGGATCCGGCAGCCACCGATGCCCTGATTGAGCAGGCCTACCGTCAGATTTACTTTCACGCCTTCAAAGTGGACCGTGATGCGGTGCTGGAATCCCAGCTGCGCTCCGGTCAGATCAACACCCGCGACTTCATCCGCCAACTCCTGCTCTCCGAGAAGTTTCAGAGGGATTTTTATCGCTGCAATAGCAATTACAGGGTGGTGGAACAGGTGGTGGGTCGGGTATTTGGCCGGCCGGTGCATGGCCAAGCCGAACAGATCGCCTGGTCGATCGTGATCGCCGAGCAGGGTCTGCCCAAGTTTGTTGACGCGCTACTGAATTCCGACGAATACCGCGACAACTTCGGCGACAACCTGGTGCCTTTTCAGCGCTCGCGGGTGCTTCCCGGCCAGGCGGTAGGCACCATGCCCTTCAACCAGCAAGCGCCTCGCTACGACTCCTACTGGCGTGAGGCGATGGCCAGGCGCGCTCCCGCTGGCGGATATCCCTGGACGGCGGGCGGCGGCTGGCCGCGCCCAGCCTGGCTGGAAGGGCAGCCCACGCCAAGGGTTCAAGCTATTTGGCAGTACACCGTGGCAACTGGGGGCTTTGTGCTCACCGGCCTGGTGATCTGGATTGCTGCGGCCATGCTC


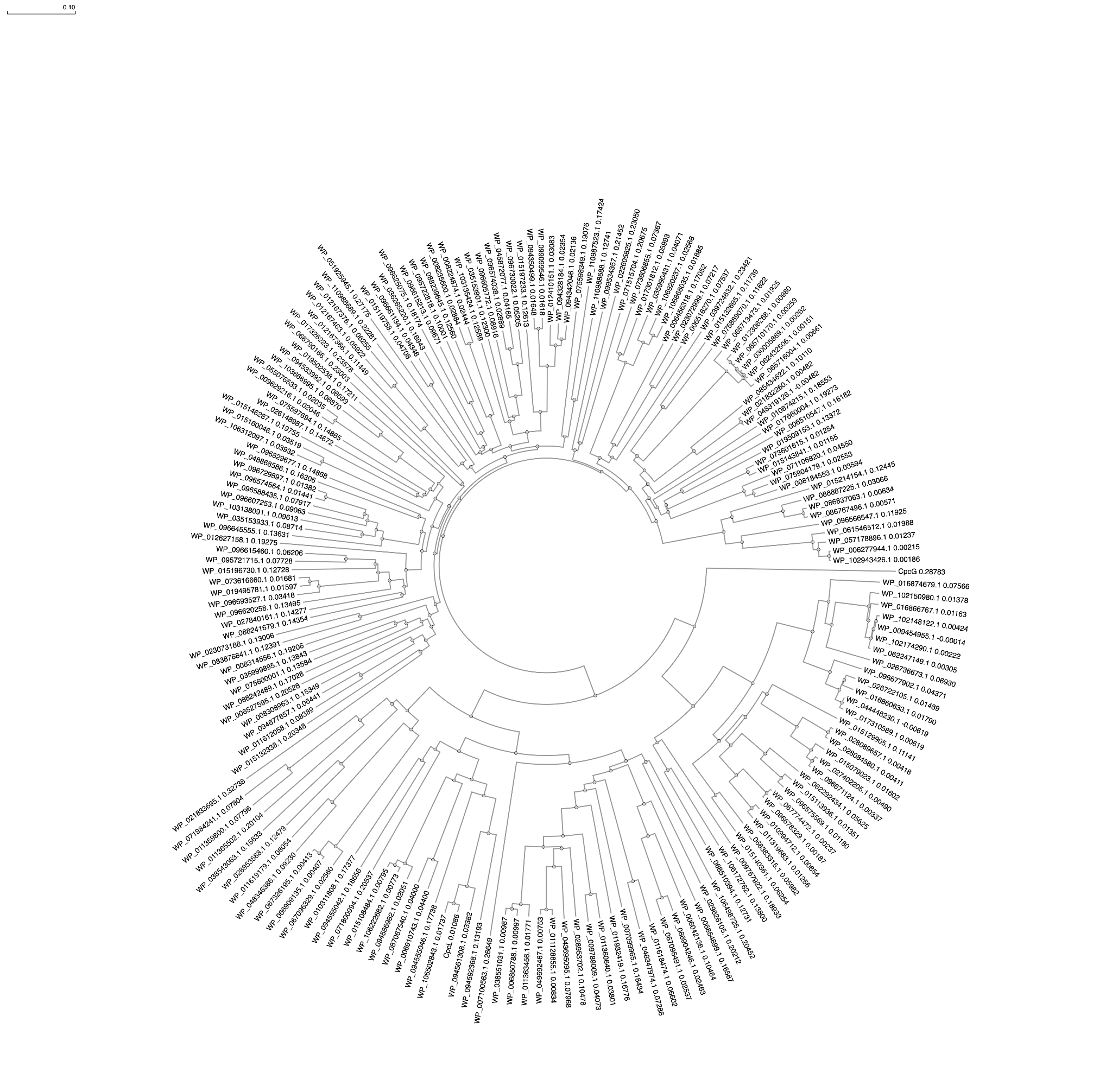


**Supplementary Figure S1**: Phylogenetic analysis of CpcL sequences. The identified sequence in the *Cyanobium* sp. NIVA-CYA 375 strain is indicated as CpcL. Multiple sequence alignment was performed using the Clustal Omega algorithm implemented at the EMBL-EBI web server. Clustal Omega employs a progressive alignment approach combined with Hidden Markov Model (HMM) profile-profile alignment. Initially, pairwise sequence similarities are estimated using k-tuple methods, followed by clustering to generate a guide tree. The final multiple sequence alignment is then constructed progressively according to this guide tree. Default parameters were used for all analyses. The multiple alignment of CpcL sequences is provided in a separate file. Phylogenetic relationships were inferred from the multiple sequence alignment using the Simple Phylogeny tool (EMBL-EBI). The guide tree produced during the alignment process was constructed based on pairwise sequence distances using the Neighbor-Joining method. The tree was rooted to the P50039 PYG1_THEVB sequences (simply marked as CpcG). Branch lengths represent evolutionary distances derived from pairwise sequence comparisons and correspond to the estimated number of substitutions per site. These values reflect genetic divergence between sequences and do not indicate statistical support (e.g., bootstrap values).


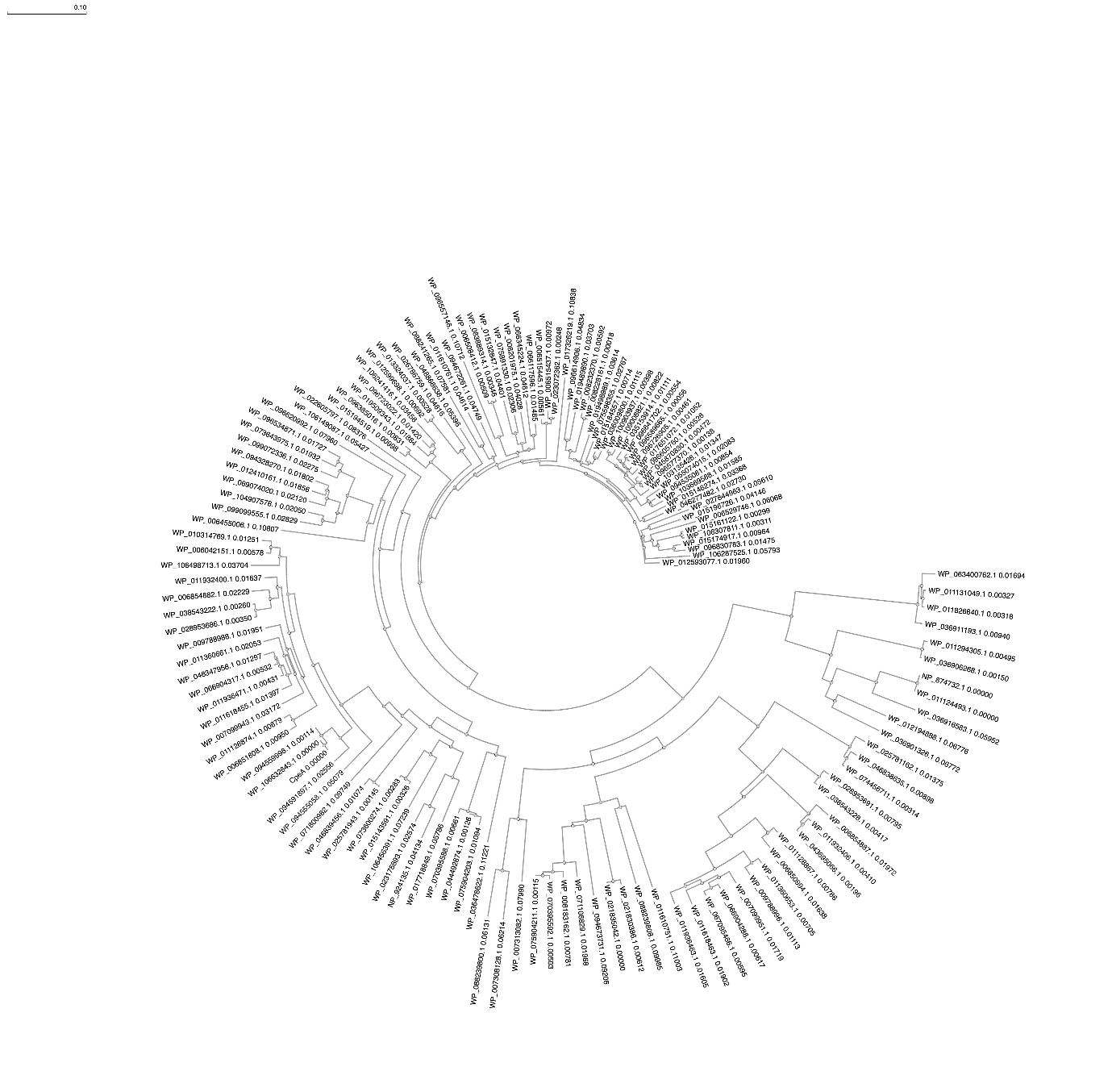


**Supplementary Figure S2**: Phylogenetic analysis of CpeA sequences. The identified sequence in the *Cyanobium* sp. NIVA-CYA 375 strain is indicated as CpeA. The method of multiple sequence alignment and construction of the phylogenetic tree was the same as presented above. The multiple alignment of CpeA sequences is provided in a separate file. Branch lengths represent evolutionary distances derived from pairwise sequence comparisons and correspond to the estimated number of substitutions per site. These values reflect genetic divergence between sequences and do not indicate statistical support (e.g., bootstrap values).


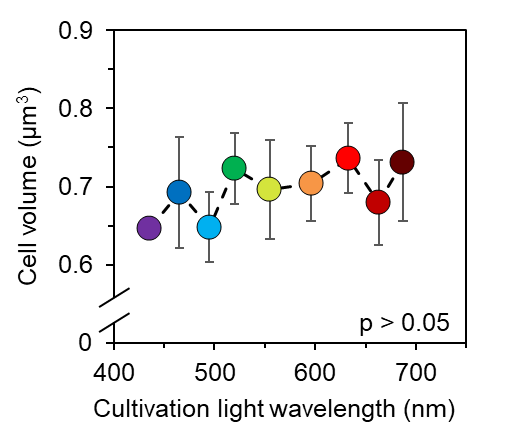


**Supplementary Figure S3**: Cell volume of *Cyanobium* cells grown under various monochromatic lights. The values represent mean ± SD (n = 3–4).


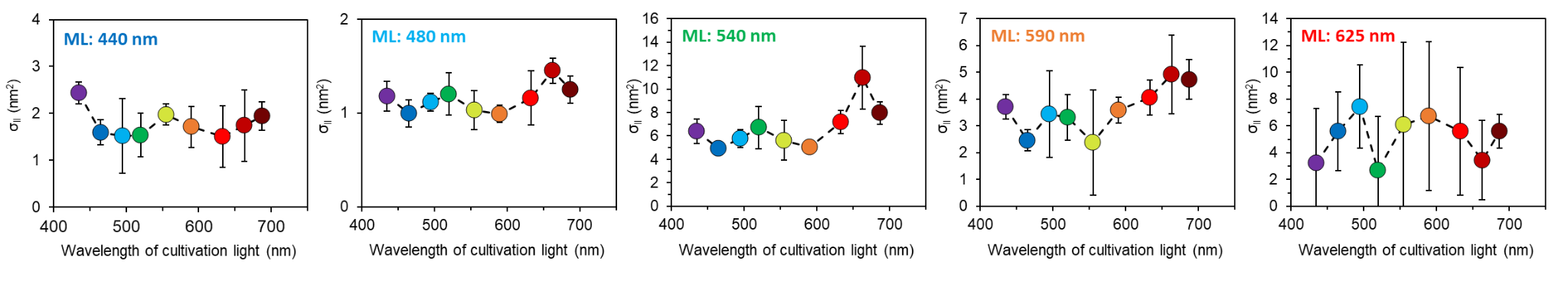


**Supplementary Figure S4**: Functional absorption cross-section of PSII (measuring lights from left to right: 440 nm, 480 nm, 540 nm, 590 nm and 625 nm) in *Cyanobium* cells cultivated under monochromatic lights. The values represent mean ± SD (n = 3–4).


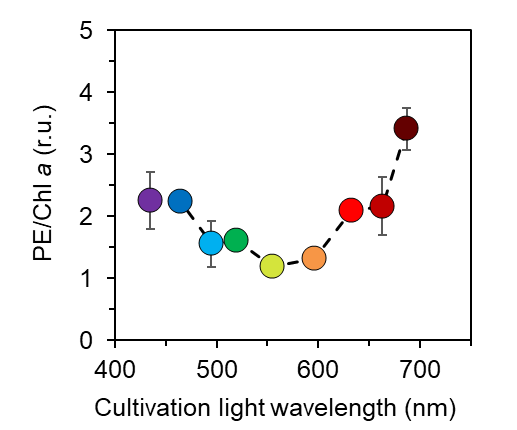


**Supplementary Figure S5**: The ratio of phycoerythrin relative to Chl *a*, based on biochemical assays. The values represent mean ± SD (n = 3–4).


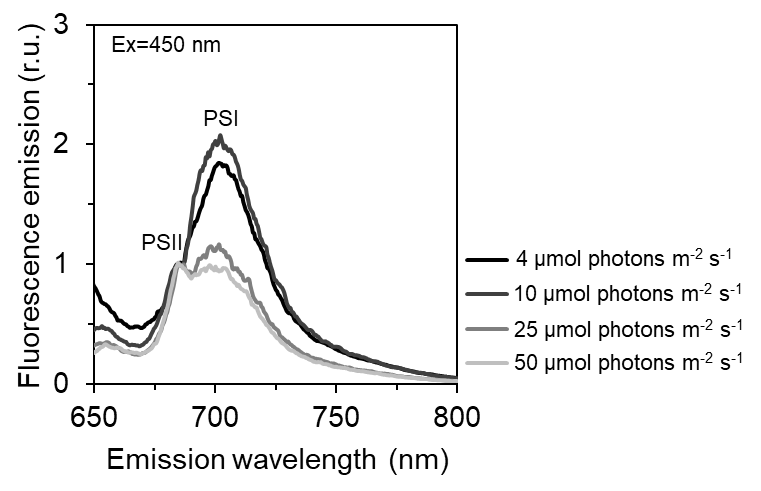


**Supplementary Figure S6**: Low temperature (77 K) fluorescence emission spectra of *Cyanobium*, upon excitation at 450 nm. The spectra were normalized to the PSII-associated peak/shoulder at 685 nm. The cultures were grown under warm white fluorescent light at different light intensities (4, 10, 25, and 50 μmol photons m^-2^ s^-1^).

**References:**

[Hirose, Yuu, Song Chihong, Mai Watanabe, et al. 2019. “Diverse Chromatic Acclimation Processes Regulating Phycoerythrocyanin and Rod-Shaped Phycobilisome in Cyanobacteria.” *Molecular Plant* 12 (8): 1167–1169.](http://paperpile.com/b/Fc16UX/VrQ6)

[Mondal, Soumila, Deepa Pandey, and Shailendra P. Singh. 2024. “Chromatic Acclimation in Cyanobacteria Renders Robust Photosynthesis and Fitness in Dynamic Light Environment: Recent Advances and Future Perspectives.” *Physiologia Plantarum* 176 (5): e14536.](http://paperpile.com/b/Fc16UX/X6rB)

[Sanfilippo, Joseph E., Laurence Garczarek, Frédéric Partensky, and David M. Kehoe. 2019. “Chromatic Acclimation in Cyanobacteria: A Diverse and Widespread Process for Optimizing Photosynthesis.” *Annual Review of Microbiology* 73 (September): 407–433.](http://paperpile.com/b/Fc16UX/VUyf)
